# Cytoreductive surgery followed by hyperthermic intraperitoneal chemotherapy applications in upper and lower gastrointestinal cancer, a review

**DOI:** 10.3389/or.2024.1496141

**Published:** 2024-11-26

**Authors:** Denise Drittone, Francesca Matilde Schipilliti, Giulia Arrivi, Federica Mazzuca

**Affiliations:** ^1^ Medical Oncology Unit, Sant’Andrea Hospital in Rome, Rome, Italy; ^2^ Medical Oncology Unit, Azienda Sanitaria Locale Roma 5, Rome, Italy; ^3^ Oncology Unit, Department of Clinical and Molecular Medicine, Azienda Ospedaliera Universitaria Sant’Andrea, Sapienza University of Rome, Rome, Italy; ^4^ PhD School in Translational Medicine and Oncology, Department of Medical and Surgical Sciences and Translational Medicine, Faculty of Medicine and Psychology, Sapienza University of Rome, Rome, Italy; ^5^ Oncology Unit, Department of Clinical and Molecular Medicine, Sant’Andrea University Hospital, Sapienza University of Rome, Rome, Italy

**Keywords:** peritoneal metastases (PM), cytoreductive surgery (CRS), hyperthermic intraperitoneal chemotherapy (hipec), gastrointestinal cancer (GI), colorectal cancer (CRC), gastric cancer (GC), appendiceal cancer, pancreatic cancer (PC)

## Abstract

Peritoneal metastases (PM) are the spread of tumor forms into the peritoneum as metastases from another organ. PM is a frequent condition in metastatic gastrointestinal cancer (colorectal, gastric, pancreatic, appendiceal, and cholangiocarcinoma); their presence confers a poor prognosis, reducing patient survival. The standard treatment consists of systemic chemotherapy according to current guidelines. In recent years, scientific evidence has shown how combined cytoreductive surgery (CRS) techniques followed by hyperthermic intraperitoneal chemotherapy (HIPEC) can improve survival in this patient population. Despite the results still obtained, using this combined technique is still under discussion. This review aims to highlight the benefits and limitations of this combined procedure, which is already widely used to treat peritoneal metastases in gynecological tumors.

## 1 Introduction

Peritoneal metastases (PM) is a common condition of gastrointestinal cancer (GI), and it is often associated with poor prognosis (1). PM has been regarded as a less common pattern of cancer metastases, particularly from the perspective of each discrete primary cancer but also collectively across the full spectrum of malignancies that can develop carcinomatosis (2). PM can occur through different mechanisms, including intraperitoneal spread by contiguity, diffusion hematogenous, lymphatic dissemination, and iatrogenic spread during surgery or bioptic procedure (3).

Today, the optimal approach with curative intent can be represented by CRS plus HIPEC. Hyperthermic intraperitoneal chemotherapy (HIPEC) is a localized chemotherapeutic treatment of PM performed after tumor cytoreduction surgery (CRS) that combines the concept of direct delivery of the chemotherapeutic agent to the peritoneum, enabling the application of higher local doses with low systemic toxicity, and the enhancement of its cytotoxic effects using hyperthermia (4). Therefore, this treatment involves a complex surgical intervention, to obtain the maximum benefit, an optimal selection of patients based on performance status and an accurate study of the extent of the disease is needed (5).

Patient selection is a crucial aspect of planning for treating PM from gastrointestinal cancer (6). Thanks to the work of the high volume centers from around the world 8, clinical and radiographic variables associated with increased chances of achieving a complete cytoreduction have been listed: Eastern Cooperative Oncology Group (ECOG) performance status <1; no evidence of extra-abdominal disease; up to three small, resectable parenchymal hepatic metastases; no evidence of biliary obstruction; no evidence of ureteral obstruction; no evidence of intestinal obstruction at more than one site; small bowel involvement: no evidence of gross disease in the mesentery with several segmental sites of partial obstruction; small volume disease in the gastro-hepatic ligament (7). Table 1 summarizes the principle clinical criteria for selecting the major possibility of treatment of PM.

**TABLE 1:** Clinical and radiographic variables associated with increased chances of achieving complete cytoreduction in patients with peritoneal metastases (PM) from gastrointestinal cancer.

Variable	Criteria
ECOG Performance Status	0 or 1
Extra-Abdominal Disease	Absent
Hepatic metastases	<3
Bilary Obstruction	Absent
Ureteral Obstruction	Absent
Intestinal Obstruction	Absent
Small Bowel Involvemet	Absent
Gastro-Hepatic Ligament Disease	Small volume disease

Clinical and laboratory factors influence the prognosis of good response to HIPEC and CRS. For patients undergoing CRS, the peritoneal cancer index (PCI) is one of the most important prognostic factors used in selecting patients for surgery that has an impact on treatment outcomes (8). PCI is a tool to evaluate the preoperative and intraoperative extent of the disease. The peritoneal cavity is divided into 13 regions, and a score from 1 to 3, depending on lesion size, is recorded for each. A final score from 1 to 39 can be used as a prognostic indicator for the disease course (9). Even if a higher PCI is associated with worse survival, it was never defined a cut-off for complete CRS and HIPEC failure (10).

Several studies have evaluated the timing of the onset of peritoneal metastases, but the results showed no differences in survival between the synchronous or metachronous presentation of peritoneal metastases. Grade III/IV morbidity was independently associated with worse overall survival. Cytoreduction (CC) completeness evaluates the most extensive residual tumor nodules. Patients with no visible residual tumor after surgical de-bulking are given a score of CC-0, while those with the most significant residual tumor nodules <2.5 mm are given CC-1 scores. CC-2 is designated for the most significant tumor deposits between 2.5 mm and 2.5 cm in size, and CC-3 is for tumors greater than 2.5 cm or confluence of multiple smaller nodules. Ideally, surgery with therapeutic intent is aimed at achieving a CC of 1 or less (11).

In addition to HIPEC there are several other approaches for treating PM. These techniques include pressurized intraperitoneal aerosol chemotherapy (PIPAC) for peritoneal cancer. PIPAC delivers chemotherapy as a pressurized aerosol into the peritoneal cavity, offering minimally invasive and targeted treatment. Initial studies show promising results in terms of drug delivery and efficacy. However, further research is needed to confirm long-term benefits, safety, and optimal patient selection (12). Intraperitoneal pre-targeted radioimmunotherapy is another therapeutic approach that combines pretargeting techniques with radioimmunotherapy to treat peritoneal carcinomatosis caused by colorectal cancer. This method involves the administration of a targeting agent that binds specifically to cancer cells, followed by a radioactive agent that attaches to the targeting agent, increasing the localized radiation exposure of cancer cells while sparing healthy tissues. Among the advantages of this approach are increased efficacy and reduced systemic toxicity (13). PRIT system currently shows promise for the treatment of patients with peritoneal epithelial ovarian carcinomatosis (EOC). Ongoing studies aim to optimize targeting and improve the therapeutic index for clinical application (14).

Park SY et al. in 2016 conducted a case-control study from a single center about early postoperative intraperitoneal chemotherapy (EPIC) for colorectal cancer patients after complete cytoreductive surgery. 30 patients undergoing EPIC showed significant improvement in 3-year overall survival (74.3% vs. 34.7%) and disease-free survival (53.0% vs. 7.5%) compared with 15 controls. Multivariate analysis identified EPIC as an independent prognostic factor for overall and disease-free survival, suggesting that EPIC is a safe and effective method to prevent peritoneal recurrence and improve outcomes in these patients (15).

In light of the above, this literature review aims to highlight the potential benefits of this combined procedure in upper and lower gastrointestinal tract tumors.

## 2 PM clinical presentation

Patients with peritoneal cancer, both primary and secondary, often present with a range of non-specific symptoms, including abdominal bloating, distension, nausea, indigestion, anorexia, weight loss, fatigue, constipation, and abdominal or back pain. Among these, abdominal distension and pain are the most commonly reported symptoms, while palpable abdominal masses and ascites are frequent clinical signs (16). The lack of specific symptoms can lead a difficult differential diagnosed in terms of primary or metastatic of peritoneum involvement and primary site origin of cancer.

## 3 Techniques of HIPEC

HIPEC perfusion lasts for 30–120 min within the peritoneal cavity, but the overall median duration in the underlying publications was 90 min. The median temperature used is between 42° and 43°. There are two fulfillment methodology categories: open abdomen and closed abdomen. The open, or “Coliseum,” technique is performed during laparotomic surgery in which the patient’s abdominal skin edges are suspended by a retractor apparatus alongside the integration of a silicon sheet to establish an open space for perfusion of the hyperthermic chemotherapy solution. This technique allows for a better distribution of the drugs across the peritoneal compartment and can determine the intractable heat loss from the chemotherapeutic solution during the procedure. The closed or laparoscopic technique is done laparoscopically at the end of CRS with subsequent infusion of the hyperthermic chemotherapy solution in the sealed abdominal compartment. Unlike the laparoscopic technique, it provides superior heat loss prevention despite the inefficient distribution of perfusion fluid. Fujimura described a semi-open technique that combines the benefits of both methodologies by minimizing heat loss and allowing a homogeneous distribution of drugs. The device used in this technique is the peritoneal cavity expander (PCE) (17). It is a complex surgical procedure with the potential for high morbidity. In HIPEC, frequently used chemotherapy agents include mitomycin C, oxaliplatin, cisplatin, and doxorubicin (18). These drugs are selected for their effectiveness in treating peritoneal carcinosis and enhancing patient outcomes.

## 4 Complications

The morbidity after the procedure may be classified into two major types: surgery-related and chemotherapy-related ones. Surgical-related morbidities include postoperative ileus, wound infection and sepsis, bleeding, thrombosis, and lung embolism. Chemotherapy may slow the wound healing process, leading to anastomotic time wasters and increase the risk of post-operative infections (19).

Cytostatic agents used for HIPEC may lead to systemic toxicities: bone marrow toxicity (leucopenia, anemia, thrombopenia,) heart, liver, or renal toxicity are more frequent ones (20). Treatment-related adverse events are classified into two groups: minor complications (grade 0–2) and major complications (grade 3–5) according to the classification system Common Terminology Criteria for Adverse Events (CTCAE version 5.0). Grade 3 are serious events, Grade 4 are life-threatening or disabling events, and Grade 5 are death-related events (21). In conclusion, it can be argued that morbidity rates after CRS and HIPEC are relatively high but comparable to other major gastrointestinal surgeries. However, in existing studies, the assessment of morbidity is not standardized and, therefore, often not comparable (9).

## 5 Indications

At the state-of-the-art HIPEC and CRS, a combined technique is indicated for the treatment of peritoneal metastases in upper and lower gastrointestinal cancer.

This review lists the indications in various gastrointestinal tumors with current studies and results.

### 5.1 Gastric cancer

Gastric cancer (GC) is the fifth most common cancer and the third most common cause of cancer death globally (20). Clinicopathological studies reveal that the overall incidence of metastases of GC on peritoneal surfaces is 30%–50%; in addition, the presence of PM is characteristic of diffuse type (45%–75% vs. 10%–30% for intestinal type) (22). For potentially resectable stage IB-III gastric cancers, with node involvement and signet/mucinous histotype, laparoscopy and peritoneal washings for malignant cells are recommended by ESMO guidelines to detect any peritoneal metastatic disease that may not be visible on imaging or macroscopically, with an overall sensitivity of 84.6% and specificity of 100% for identifying peritoneal metastases (23). The NCCN gastric cancer guidelines classify positive peritoneal cytology (CY+) as metastatic (M1) disease and suggest palliative treatments (24).

Literature data is favoring using CRS plus HIPEC to improve the outcome of patients with PM from GC (25). A multicenter, randomized controlled trial (RCT) (GASTRIPEC‐I‐trial, NCT02158988) exploring the impact of HIPEC after CRS on survival, showed a similar median survival (14.9 vs. 14.9 months, *p* = 0.16) when compared to the CRS‐only arm; however, both the progression‐free survival (7.1 vs. 3.5 months P = 0.047) and metastases‐free survival (10.2 vs. 9.2 months; P= 0.0286) were significantly improved in the combination arm (26). At the 2024 Fata update, the study showed no OS difference between the CRS + HIPEC and CRS-only arms. PFS and MFS were significantly better in the CRS + H group, which needs further exploration. HIPEC did not increase the frequency of grade ≥3 AEs (27). No solid evidence shows improved survival in gastric cancer patients after CRS-HIPEC treatment (18). Nevertheless, an improvement in survival outcomes has been observed with CRS-HIPEC over CRS alone (11.2 months vs. 5.6 months) in a phase III randomized trial [PMID: 21431408] (25).

### 5.2 Colorectal cancer

Colorectal cancer (CRC) is the third most common malignancy and the second leading cause of cancer-related mortality in the world (28). The peritoneum is one of the most common metastatic sites in CRC, along with the liver and lung, and compared to other sites of disease, peritoneal metastases present a worse prognosis (29). It is estimated that the peritoneum is the only site of metastases in 25% of patients with CRC (30). PM in CRC patients are linked to significantly poor prognostic outcomes (31). With modern systemic chemotherapy combinations, including oxaliplatin and irinotecan along with 5-FU and targeted agents, the median OS for patients with stage IV colorectal cancer has improved significantly, now ranging from approximately 7 to over 24 months (32). In particular, in patients with PM alone, a median survival of 9 months was reported when treated with modern systemic chemotherapy alone (6). Peritoneal treatments can have positive effects on survival, offering significant benefits in selected patients, however, it is essential to emphasize that in the context of colon cancer, systemic chemotherapy (CT) remains crucial for disease control and improving long-term outcomes (33).

Regarding the potential beneficial effect on the survival of HIPEC, until now, only one randomized study has been conducted, a multicentric French trial (PRODIGE 7 trial, NCT00769405), which compared CRS plus HIPEC to CRS without HIPEC. The PRODIGE 7 trial showed that adjuvant HIPEC did not improve recurrence-free and overall survival in this relatively small cohort of patients. The trial established the value of high-quality CRS in both arms of the trial, with a higher-than-expected median overall survival of 41 months in both groups (34).

A multicenter study published by Fisher O.M. in 2024 assessed the efficacy of HIPEC in patients with colorectal cancer and peritoneal metastases (pmCRC) using a large international dataset. It involved 2,093 patients from 39 centers who underwent cytoreductive surgery with HIPEC between 1991 and 2018, comparing two HIPEC protocols: oxaliplatin-HIPEC and mitomycin-HIPEC. The oxaliplatin-HIPEC group had a significantly longer OS (47 months) than the mitomycin-HIPEC group (39 months). Notably, the combination of oxaliplatin and irinotecan in HIPEC yielded the best OS (61 months). The study concluded that oxaliplatin-based HIPEC provides superior outcomes compared to mitomycin-based HIPEC, with lower 90-day mortality rates observed in the oxaliplatin group and a trend toward a dose-response relationship (35).

In Table 2 are listed ongoing trials on HIPEC in CRC and GC in the metastatic setting.

**TABLE 2:** Ongoing trials in the metastatic setting.

NCT Number/Trial name	Trial Design	Characteristic of patients enrolled	Number of pts	Primary endpoint
GASTRIPEC I (NCT02158988)	Phase 3 study	GC/GEJ with peritoneum metastases without evidence of other metastatic sites treated with neoadjuvant CT followed HIPEC + postoperative chemotherapy (Group B) GC/GEJ with peritoneum metastases without evidence of other metastatic sites treated with cytoreduction alone after neoadjuvant CT and postoperative CT (Group A)	105	OS
PRODIGE 7 (NCT00769405)	Phase 3 trial	CRC with complete macroscopic resection or surgical resection with less than 1 mm residual tumour tissue was completed were randomly assigned (1:1) to cytoreductive surgery with or without oxaliplatin-based HIPEC	265	OS

Abbreviations: GC, gastric cancer; GEJ, gastro oesophageal junction cancer; CT, chemotherapy; HIPEC, hyperthermic intraperitoneal chemotherapy; CRC, colorectal cancer; OS, overall survival.

### 5.3 Appendiceal cancer

Appendiceal tumors are rare and constitute less than 0.5% of all neoplasms of gastrointestinal origin (36). Appendiceal cancer can potentially disseminate into the peritoneal cavity, causing PM in approximately 20% of patients (37). Glehen et al., in their retrospective multicenter study of >500 patients, suggested clearly that the therapeutic approach combining cytoreductive surgery with peri-operative HIPEC had significantly better long-term survival than patients who did not. Patients in whom cytoreductive surgery was complete had a median survival of 32.4 months, compared with 8.4 months for patients in whom complete cytoreductive surgery was impossible (*p* < .001). The outcomes results are comparable to colorectal cancer but not significantly better as might have been expected in appendiceal cancer (38).

### 5.4 Pancreatic cancer

Pancreatic cancer (PC) is a relatively rare and lethal disease with incidences ranging from 6.4 to 7.7 cases per 100,000 people per year in Western countries. It is the 7th most common cause of cancer-related mortality (39). However, many patients are diagnosed at an advanced stage, and the peritoneum is the second most common site of metastases in the case of disseminated pancreatic disease (40). The presence of PM worsens its prognosis. There is currently no broad consensus for the use of intraperitoneal infusion of chemotherapeutic substances in the treatment of pancreatic cancer. HIPEC could be considered a promising technique for improving survival rate without additional morbidity in case of borderline resectable and locally advanced disease when surgical resection and CRS are possible after neoadjuvant treatment (41). There is a phase II study currently enrolling patients (NCT04858009) (42); the trial studies the effects of HIPEC in the treatment of patients with pancreatic cancer with peritoneal metastases. This study may help doctors determine the safety and effectiveness of HIPEC in treating pancreatic cancer patients. The primary objectives of the trial are OS and DFS.

### 5.5 Cholangiocarcinoma

Cholangiocarcinoma is an epithelial cell malignancy arising from varying locations within the biliary tree, and it is divided intrahepatic, perihilar, and distal (43). Tumors of the biliary tract account for 1% of all cancers and approximately 10%–15% of all primary cancers originating in the liver (44). The peritoneum is the most frequent site of metastases in cholangiocarcinoma (45) 10%–20% of patients have peritoneal involvement at presentation (46). In 2021, Feng et al. performed a retrospective study to compare the prognosis of patients with advanced intrahepatic cholangiocarcinoma (ICC) undergoing CRS + HIPEC *versus* CRS alone (47). However, prospective studies are required to validate these findings further and support the implementation of this technique in clinical practice. The median OS was longer in the CRS + HIPEC group than in the CRS group (25.53 vs. 11.17 months, *p* < 0.001). CRS + HIPEC could be a treatment option for patients with advanced ICC, with improved OS and similar complications and adverse events compared with CRS alone.

## 6 Recurrence prevention

Several clinical trials are underway to evaluate the effectiveness of HIPEC in reducing peritoneal recurrence and improving survival outcomes when combined with CRS.

The GASTRICHIP study is a prospective, open-label, randomized multicenter phase III clinical trial designed to evaluate the effects of HIPEC with oxaliplatin in patients with GC that involves the serosa and/or lymph node metastases, or positive cytology in peritoneal washing. The trial will include patients scheduled for D1-D2 curative gastrectomy. Primary outcome is OS measured from the date of surgery to the date of death or the end of the 5-year follow-up period; secondary outcomes are efficacy assessed through 3-year and 5-year recurrence-free survival rates, localization of recurrence, morbidity, and quality of life (48). The PERISCOPE II trial assesses the efficacy of combining gastrectomy, CRS, and HIPEC for patients with gastric cancer that has limited peritoneal dissemination. This multicenter randomized controlled trial aims to determine if this treatment approach provides a survival benefit compared to standard palliative systemic chemotherapy. The primary outcome measured will be overall survival, with secondary outcomes focusing on complications and quality of life. The study began in October 2017 and is expected to complete by 2029 (49). Regarding the adjuvant setting in CRC, the COLOPEC trial investigated the efficacy of adjuvant HIPEC in patients with locally advanced CRC after cytoreductive surgery. Participants with clinical or pathological T4 N0-2 M0 or perforated colon cancer were randomly assigned to receive either adjuvant systemic chemotherapy with HIPEC or adjuvant systemic chemotherapy alone. After a median follow-up of 59 months, the results showed no significant differences in the 5-year overall survival rate (69.6% vs. 70.9%), peritoneal metastases rates (63.9% vs. 63.2%), or disease-free survival (55.7% vs. 52.3%) between the two groups. Additionally, quality-of-life outcomes were similar. The findings suggest that adjuvant HIPEC should be reserved for clinical trials rather than standard practice (50). The PROPHYLOCHIP trial examined the benefit of systematic second-look surgery combined with HIPEC in colorectal cancer patients at high risk for PM. Patients who remained recurrence-free post-surgery and chemotherapy were randomized to surveillance or a treatment group receiving HIPEC with oxaliplatin. Results showed no significant difference in 3-year disease-free survival, peritoneal recurrence-free survival, or overall survival. High postoperative complications and potential oxaliplatin resistance were noted, raising concerns about HIPEC’s effectiveness in this setting (51).

The HIPECT4 trial was a phase 3 randomized controlled trial conducted in 17 centers across Spain from 2015 to 2021. It aimed to evaluate the efficacy of HIPEC with mitomycin C in patients with T4 colon cancer, focusing on locoregional disease control. Patients were randomized to receive either CRS with HIPEC followed by systemic chemotherapy or CRS alone with systemic chemotherapy. After 3 years, the locoregional disease control rate was significantly higher in the HIPEC group (97.6%) compared to the control (87.6%), with no differences in disease-free or overall survival (51). Table 3 lists ongoing studies in the adjuvant setting.

**TABLE 3:** Ongoing trials in the adjuvant setting.

NCT Number/Trial name	Trial Design	Characteristic of patients enrolled	Number of pts	Primary endpoint
GASTRICHIP (NCT01882933)	Phase 3 trial	GC T3, T4 and/or N+ and/or with positive peritoneal cytology. Randomization in two arms: Arm A: curative gastrectomy with D1-D2 lymph node dissection + HIPEC with oxaliplatin Arm B: curative gastrectomy with D1-D2 lymph node dissection	367	OS
PERISCOPEII (NCT03348150)	Phase 3 trial	GC with peritoneal dissemination. Randomization between gastrectomy + cytoreductive surgery + HIPEC (experimental arm) and palliative systemic chemotherapy (standard arm)	182	OS
COLOPEC (NCT02231086)	Phase 3 trial	CRC with T4 or intra-abdominally perforated in preventing the development of peritoneal carcinomatosis in addition to the standard adjuvant systemic CT *versus* CT only	204	RSF
PROPHYLOCHIP (NCT01226394)	Phase 3 trial	CRC at high risk of developing PM after resection of primary tumor after 6 months of adjuvant CT without evidence of recurrence. Surveillance-only randomization vs. second-level surgery with CRS-HIPEC	130	3 years DFS
HIPECT4 (NCT02614534)	Phase 3 trial	CRC T4N02M0. Randomization between CRS only vs. concurrent CRS–HIPEC	200	LC

Abbreviations: GC, gastric cancer; GEJ, gastro oesophageal junction cancer; CT, chemotherapy; HIPEC, hyperthermic intraperitoneal chemotherapy; CRC, colorectal cancer; OS, overall survival; RSF, recurrence free survival; DFS, disease free survival; LC, locoregional control.

## 7 Discussion

Peritoneal metastases (PM) occur when tumor cells spread to the peritoneum from another organ, frequently arising from gastrointestinal cancers. The presence of PM generally suggests a poor prognosis and significantly reduces patient survival, leading to exclusive systemic chemotherapy. However, recent studies suggest that combining CRS with HIPEC and systemic chemotherapy may improve survival outcomes for patients with PM.

Research on the application of CRS combined with HIPEC is evolving, particularly concerning its integration with emerging cancer therapies.

Fisher O.M. et al showed that oxaliplatin-HIPEC significantly improved overall survival in pmCRC. In addition, Feng et al. in 2021 found that SRC + HIPEC improved median overall survival in patients with advanced intrahepatic cholangiocarcinoma (ICC) compared with SRC alone (25.53 vs. 11.17 months), although further prospective studies are needed.

An innovative field of research in PM management is represented by the application of nanoparticles to improve the efficacy of HIPEC. Nanoparticles possess unique properties that make them suitable for drug delivery, including a high surface-to-volume ratio, the ability to encapsulate therapeutic agents, and the potential for targeted delivery to tumor sites (52). Indeed, Tang L et al. in 2021, highlighted the unique properties of nanoparticles that allow for improved drug solubility, stability, and targeted delivery to tumor sites. Integrating nanoparticles in these therapies can enhance treatment efficacy while minimizing systemic side effects. However, toxicity, safety, and regulatory hurdles must be addressed (53).

Another intriguing field to enhance the HIPEC efficacy, is represented by the microenvironment effect on response to treatment of PM. While HIPEC enhances immune responses by promoting T- and NK-cell infiltration and increasing antigen presentation, it can also induce immunosuppressive changes, such as increased regulatory T cells and myeloid-derived suppressor cells. This complexity requires an individualized immune profile and suggests that combining HIPEC with immunotherapies may optimize treatment outcomes for patients with peritoneal carcinosis. The balance between pro- and anti-tumor immunity is crucial to improve prognosis (54). In particular, catumaxomab, a trifunctional antibody targeting EpCAM, has been shown to reduce malignant ascites. Intraperitoneal immunotherapy aims to enhance T-cell responses and includes treatments such as CAR-T cells that can enhance immune activity against tumors expressing carcinoembryonic antigen (55). The ImmunoPeCa trial (NCT02219893) was a phase 1 study assessing the safety of the MOC31PE immunotoxin in 21 colorectal cancer patients with peritoneal metastases following CRS/HIPEC. It was conducted in 2017 and found MOC31PE to be safe and well-tolerated, with cytotoxic levels in peritoneal fluid despite limited systemic absorption. Neutralizing antibodies were produced in all patients, leading to a 3-year overall survival estimate of 78% and a median progression-free survival of 21 months, indicating the need for further evaluation of MOC31PE’s effectiveness (56).

Continued exploration in this field may enhance treatment outcomes for patients with peritoneal carcinosis.

## 8 Conclusion

The management of PM in gastrointestinal cancers presents a significant challenge in oncology due to the historically poor prognosis associated with this condition. While systemic chemotherapy has been the conventional treatment until nowadays, recent advancements in surgical techniques and localized therapies as HIPEC, offer promising alternatives to achieve the survival outcomes. Although randomized trial evidence remains non-homogeneous, the integration of CRS with HIPEC seems to provide progression-free and metastases-free survival advantages, especially among highly select patient populations.

Nevertheless, the complexity of the procedure underscores the importance of careful patient selection and a comprehensive understanding of the disease’s extent to optimize outcomes. As research progresses, particularly with the investigation of innovative drug delivery systems, there is a significant opportunity to enhance the efficacy of HIPEC. Considering the dual influence of HIPEC on tumor’s immune environment, the recognition of immune profile of microenvironment could represent a possibility to personalize the therapeutic approach of PM. Future studies should concentrate on refining treatment protocols, standardizing assessments of morbidity, and exploring combination therapies to ensure that patients with peritoneal metastases receive the most effective and individualized care possible.
